# Insights into the Emergent Bacterial Pathogen *Cronobacter* spp., Generated by Multilocus Sequence Typing and Analysis

**DOI:** 10.3389/fmicb.2012.00397

**Published:** 2012-11-22

**Authors:** Susan Joseph, Stephen J. Forsythe

**Affiliations:** ^1^Pathogen Research Group, School of Science and Technology, Nottingham Trent UniversityNottingham, UK

**Keywords:** *Cronobacter*, MLST, MLSA, sequence typing, ST4

## Abstract

*Cronobacter* spp. (previously known as *Enterobacter sakazakii*) is a bacterial pathogen affecting all age groups, with particularly severe clinical complications in neonates and infants. One recognized route of infection being the consumption of contaminated infant formula. As a recently recognized bacterial pathogen of considerable importance and regulatory control, appropriate detection, and identification schemes are required. The application of multilocus sequence typing (MLST) and analysis (MLSA) of the seven alleles *atpD*, *fusA*, *glnS*, *gltB*, *gyrB*, *infB*, and *ppsA* (concatenated length 3036 base pairs) has led to considerable advances in our understanding of the genus. This approach is supported by both the reliability of DNA sequencing over subjective phenotyping and the establishment of a MLST database which has open access and is also curated; http://www.pubMLST.org/cronobacter. MLST has been used to describe the diversity of the newly recognized genus, instrumental in the formal recognition of new *Cronobacter* species (*C. universalis* and *C. condimenti*) and revealed the high clonality of strains and the association of clonal complex 4 with neonatal meningitis cases. Clearly the MLST approach has considerable benefits over the use of non-DNA sequence based methods of analysis for newly emergent bacterial pathogens. The application of MLST and MLSA has dramatically enabled us to better understand this opportunistic bacterium which can cause irreparable damage to a newborn baby’s brain, and has contributed to improved control measures to protect neonatal health.

## Introduction

*Cronobacter* spp. (formerly known as *Enterobacter sakazakii*) is a genus consisting of Gram negative, motile, facultatively anaerobic opportunistic bacterial pathogens belonging to the Enterobacteriaceae family (Kucerova et al., 2011). The diverse genus accommodates seven species: *C. sakazakii*, *C. malonaticus*, *C. turicensis*, *C. muytjensii*, *C. dublinensis*, and the two newly defined species, *C. universalis* and *C. condimenti* (Joseph et al., 2012a). The primary niche of this organism is believed to be plant material (i.e., wheat, rice, herbs, and spices; Iversen and Forsythe, 2003). However, it is brought into contact with humans via food and environmental exposure. It has been isolated from a wide range of foods including cereals, rice, cheese, fruits, meat, milk, vegetables, grains, herbs, and spices as well as their by-products (Iversen and Forsythe, 2003; Friedemann, 2007). *Cronobacter* spp. have been isolated from other mammals and invertebrates such as rats and flies (Gakuya et al., 2001; Mramba et al., 2006).

Kucerova et al. (2010) were the first to publish a detailed genome description for *Cronobacter*. The sequenced strain, *C. sakazakii* BAA-894, had been isolated from formula powder. This was later followed by the announcement of two further genomes, *C. sakazakii* E899 and *C. turicensis* z3032, though without detailed descriptions (Stephan et al., 2010; Chen et al., 2011). Kucerova et al. (2010, 2011) in two detailed publications used whole genome sequence analysis of *C. sakazakii* strain BAA-894 and microarray based comparative genomic hybridization (CGH) to explore the genomes of strains across the *Cronobacter* genus. These strains had been chosen using MLST to ensure they represented the diverse genus. They identified several variable regions which are putative virulence factors, i.e., fimbriae and multidrug efflux systems, many of which are plasmid borne (Franco et al., 2011). Putative virulence traits of particular interest are iron-uptake mechanisms (Grim et al., 2012), superoxide dismutase (SodA) for macrophage survival (Townsend et al., 2008), hemolysin (Cruz et al., 2011a), flagella (Cruz et al., 2011b), pili, a metalloprotease (Kothary et al., 2007), an enterotoxin (Pagotto et al., 2003), and plasmid borne virulence factors such as *Cronobacter* plasminogen activator (Cpa) and type six secretion systems (T6SS; Franco et al., 2011).

The majority of reported *Cronobacter* cases are in adults (FAO/WHO, 2008); however neonates and infants are the major identified group at risk due to the associated high mortality rate following necrotizing enterocolitis (NEC), septicemia, and meningitis. In neonatal cases of *Cronobacter* meningitis, there is gross destruction of the brain, leading sadly to either death (40–80% of cases) or severe neurological damage. Due to the understandable sensitivity toward neonatal infections, such cases have attracted more attention than infections in other age groups. *Cronobacter* can attach to intestinal cells, invade, and survive in macrophages (Townsend et al., 2007, 2008). OmpA and OmpX possibly have a role in the organism penetrating the blood brain barrier. The reason for the destruction of the brain cells is unknown and could, in part, be a host response (Kim et al., 2010). Infections in older age groups are principally bacteremias as well as urosepsis and wound infections. To date, only strains from the three species *C. sakazakii*, *C. malonaticus*, and *C. turicensis* have been associated with neonatal infections (Joseph et al., 2012b). *C. malonaticus* appears to be more associated with adult than neonatal infections (Joseph and Forsythe, 2011). Therefore pathogenicity in humans may be an acquired trait in this genus.

A number of neonatal outbreaks and cases of *Cronobacter* spp. infections have been reported in intensive care units (van Acker et al., 2001; Block et al., 2002; Himelright et al., 2002; Caubilla-Barron et al., 2007). Many of these infections have been directly linked to reconstituted powdered infant formula (PIF) which may have been contaminated intrinsically or during preparation and administration (Himelright et al., 2002). A common feature in some of these outbreaks is the opportunity for temperature abuse of the prepared feed, which would permit bacterial growth (Caubilla-Barron et al., 2007). It is pertinent to note that there is also asymptomatic human carriage of the organism. The bacterium has been isolated from the tracheae and feces, and additionally has been recovered from the feeding tubes of neonates fed breast milk or ready-to-feed formula, and not infant formula (Hurrell et al., 2009). Therefore, it is important that robust and reliable typing schemes are readily available for *Cronobacter* spp., and that a wide range of possible sources of the organism during an outbreak need to be investigated.

The *Cronobacter* genus belongs to the bacterial class Gammaproteobacteria, and is within the family Enterobacteriaceae. The genus is closely related to the genera *Enterobacter* and *Citrobacter*, and some *Enterobacter hormaechei* and *E. ludwiggii* strains have been mis-identified as *Cronobacter* which has led to some confusion in the literature. 16S rDNA sequence diversity has been used to define genus (5%) and species (3%) boundaries in the Enterobacteriaceae. However the method has limitations with closely related species due to minimal sequence diversity. Additionally, the sequence diversity between multiple copies of the 16S rDNA operon within a bacterium can also introduce discrepancies (Acinas et al., 2004).

Initially the *Cronobacter* genus was composed of *C. sakazakii*, *C. turicensis*, *C. muytjensii*, and *C. dublinensis* (Iversen et al., 2007). This was quickly revised (Iversen et al., 2008) with the addition of *C. malonaticus*. This species had originally been described as a subspecies of *C. sakazakii* by Iversen et al. (2007) who could not distinguish *C. sakazakii* and *C. malonaticus* using 16S rDNA sequence analysis. The *Cronobacter* species were initially differentiated by Iversen et al. (2008) according to 16 *E. sakazakii* biotypes; *C. sakazakii* (biotypes 1–4, 7, 8, 11, and 13), *C. malonaticus* (biotypes 5, 9, and 14), *C. turicensis* (biotypes 16, 16a, and 16b), *C. muytjensii* (biotype 15), and *C*. *dublinensis* (biotypes 6, 10, and 12). In contrast, Joseph et al. (2012a) used strains selected by multilocus sequence analysis (MLSA; Baldwin et al., 2009) as representatives across the genus and therefore overcame the preconceived grouping of strains based on phenotyping. These recent studies, which will be described in more detail below, led to the naming of two new *Cronobacter* species, *C. universalis* and *C. condimenti*, by Joseph et al. (2012a). Due to numerous limitations such as subjectivity and reproducibility, the earlier phenotyping approach to *Cronobacter*, based on 10 biochemical and physiological tests, has been replaced by various DNA based techniques. Consequently, biotyping no longer has a role in designating the species of *Cronobacter* isolates.

## Multilocus Sequence Typing Scheme for *Cronobacter* spp.

A number of DNA based methods for identification, speciation, and profiling have been proposed for *Cronobacter* spp. These include PCR probes for *dnaG*, *rpsU*, and *rpoB* genes (Seo and Brackett, 2005; Stoop et al., 2009). However they suffer in that they have either have not continued in use or have not been validated against a robust *Cronobacter* strain collection of the seven species. Some require different PCR primer pairs for each species. The most advanced non-MLST profiling method is serogrouping using PCR (Jarvis et al., 2011; Sun et al., 2011). However currently the method, using seven primer pairs, only covers four of the seven species and 48/231 (>20%) strains do not give a PCR product (Jarvis et al., 2011; Sun et al., 2011) which indicates that further unrecognized serogroups exist. Finally, there are contradictions in the literature with the same serogroup being across more than one species – *C. sakazakii* O3 and *C. muytjensii* O1 (Jarvis et al., 2011). In contrast to these methods, multilocus sequence typing (MLST) based on only seven primer pairs has been established for the whole *Cronobacter* genus which is robust and reliable. This article concerns the advances in our understanding of *Cronobacter* using the seven loci scheme, both for profiling and phylogeny. The MLST and MLSA approaches have:

1. Revealed the diversity of the *Cronobacter* genus.
2. Contributed to the recognition of two new species; *C. universalis* and *C. condimenti*.
3. Shown the close relatedness and groupings of the seven species.
4. Shown the evolutionary descent of the genus.
5. Revealed the majority of neonatal meningitis cases are being attributable to one clonal lineage, *C. sakazakii* ST4 clonal complex.
6. Formed the basis for future research regarding stain selection for investigating *Cronobacter* virulence and environmental fitness.
7. Established an open access curate database http://www.pubMLST.org/cronobacter. This is composed of ∼400 MLST profiled strains which are widely geographically, temporally, and sourced distributed, describing experimental protocols, contains DNA sequences of the seven alleles for offline analysis, and where users can determine the sequence type (ST) of their strains and undertake further advanced DNA sequence analysis. The database is curated (by S. Forsythe), and hosted at PubMLST by the University of Oxford, UK.

The *Cronobacter* MLST scheme requires the partial sequence analysis of seven housekeeping genes: *atpD*, *fusA*, *glnS*, *gltB*, *gyrB*, *infB*, and *ppsA* (Baldwin et al., 2009). These loci are distributed around the *Cronobacter* genome (Kucerova et al., 2010), and are under the least influence of any possible selective evolutionary pressures. Also, the primers for the seven genes have been designed to have similar annealing temperatures, enabling all reactions to be performed in a single run, thus reducing the time involved. Comparing the loci DNA sequences with the *Cronobacter* MLST reference database (http://www.pubMLST.org/cronobacter) generates the seven digit allele code, and the strain’s ST. When concatenated together the seven allele sequences form a 3036 nt length for MLSA and phylogenetic analysis.

The MLST scheme was announced first for *C. sakazakii* and *C. malonaticus* (Baldwin et al., 2009) to demonstrate its usefulness over 16S rDNA sequence analysis, which could not distinguish between them. Further publications have demonstrated the use of MLST for all seven formally recognized species of *Cronobacter* genus (Czerwicka et al., 2010; Kucerova et al., 2010, 2011; Hamby et al., 2011; Joseph and Forsythe, 2011; Hariri et al., 2012; Joseph et al., 2012a,b). The scheme has also shown the reliability of *fusA* as a single locus for speciation (Joseph et al., 2012b).

The significant contribution of MLSA and MLST to our understanding of the *Cronobacter* genus is covered later. Nevertheless, suffice it for now to point out that the initial use of biotypes (phenotyping) to support the description of *Cronobacter* species was unfortunately flawed as some biotype index strains were attributed to the wrong *Cronobacter* species, as shown by the DNA sequence based MLSA (Baldwin et al., 2009).

## Phylogeny and Evolution of the *Cronobacter* Genus

By concatenating the seven loci sequences together (3036 base pair total length) the DNA sequences can be used to construct a phylogenetic tree of the genus. This enables the quantification of intraspecific and interspecific diversity of the genus, as well as potential characterization of the strains according to virulence groupings and source.

**Figure 1:**
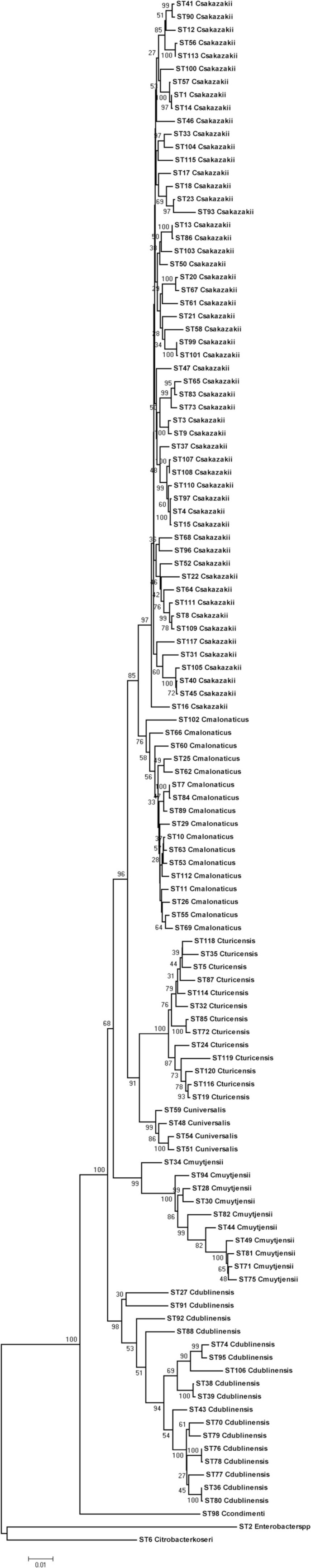
**Maximum likelihood tree based on the concatenated sequences (3036 bp) of the seven LST loci for the genus *Cronobacter***. The STs and the corresponding species are indicated at the tip of each branch. The tree is drawn to scale using MEGA5, with 1000 bootstrap replicates.

Using the Maximum Likelihood algorithm in MEGA5 (Tamura et al., 2011), one can, see the clear clustering of the various *Cronobacter* species within the genus (Figure 1). It is advisable when doing this analysis with newly profiled strains to include closely related organisms as outliers. We use the corresponding seven MLST loci sequences from the publicly available genomes of *Citrobacter koseri* (Accession number CP000822) and *Enterobacter* spp. 683 (Accession number CP000653). These two organisms are closely related to *Cronobacter* spp. yet none of their alleles are shared with any of the *Cronobacter* species (Figure 1). The reason for stressing the use of outliers is that we often receive queries from MLST users regarding problematic strains. These invariably are strains of *Enterobacter* spp. and less often *Pantoea* spp. which have been mis-identified as *Cronobacter*, or *vice versa*. These strains frequently have been provisionally identified by phenotyping and even MALDI-TOF. Unfortunately, both methods have inaccuracies in their databases.

**Figure 2:**
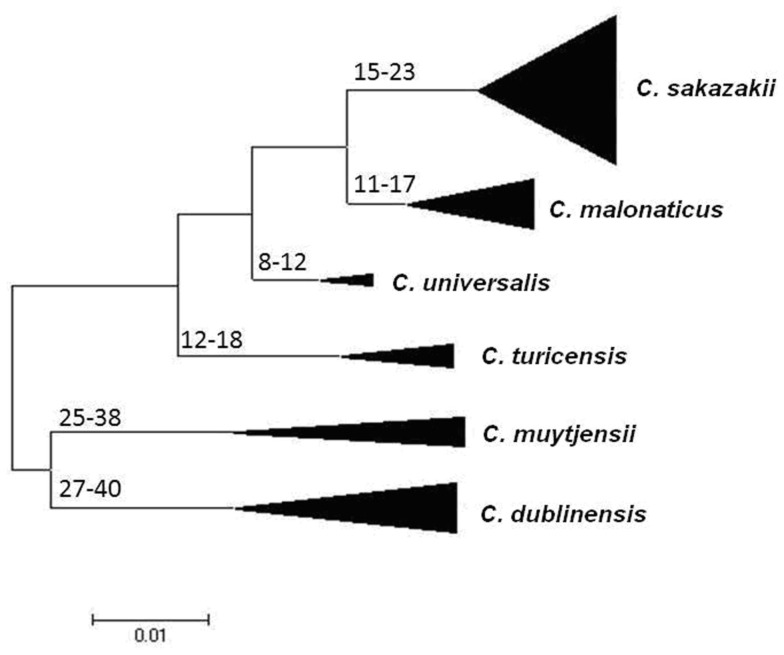
**Maximum Likelihood tree of the *Cronobacter* MLST dataset indicating ranges of the hypothetical divergence dates of each species node measured in millions of years before the present**. The tree has been drawn to scale using MEGA5. The bases of the triangles indicate the number of isolates used for the analysis, while the heights indicate the diversity of each branch. *C. condimenti* with the single isolate has been excluded from this analysis. Reproduced from Joseph et al. (2012b).

Joseph et al. (2012b) undertook an evolutionary analysis of the genus by using the mean synonymous substitution values (*D*_s_) of the *Cronobacter* MLST dataset, and previously published substitution rates for other Enterobacteriaceae members such as *E. coli* and *Salmonella*. This predicted that the *Cronobacter* genus split from its closest ancestor in the Enterobacteriaceae family approximately 45–68 million years ago (Figure 2). Accepting that such calculations are based on a number of assumptions, it is notable that this corresponds with the Paleogene period of the Cenozoic era when early flowering plants evolved. This is an interesting observation as it coincides with the suspected natural plant habitat of the organism and opens speculation for the evolution of the genus. The feeding of insect larvae on plants could have led to a host adaptation. It is notable that *Cronobacter* have been isolated from flies (Mramba et al., 2006; Pava-Ripoll et al., 2012). The evolution of other members of the Enterobacteriaceae family has been estimated by similar MLST studies and hence comparable. The evolution of the distinguishable *Cronobacter* species appears to have occurred over the same period as the divergence of the *Salmonella* species and subspecies, after its split from *E. coli* (McQuiston et al., 2008). Further analysis using whole genomes will be more informative.

The estimates of the divergence dates of the individual species have been calculated. The earliest branches of the genus led to *C. dublinensis* and *C. muytjensii* (25–40 MYA), more recent divergences have led to *C. malonaticus* and *C. universalis* (8–17 MYA). *C. condimenti* has to date been excluded from this analysis because only one isolate has been identified for this species, and hence it was not possible to calculate the necessary *D*_s_ values for the species (Joseph et al., 2012b).

## Splitstree Analysis for Homologous Recombination Visualization

**Figure 3:**
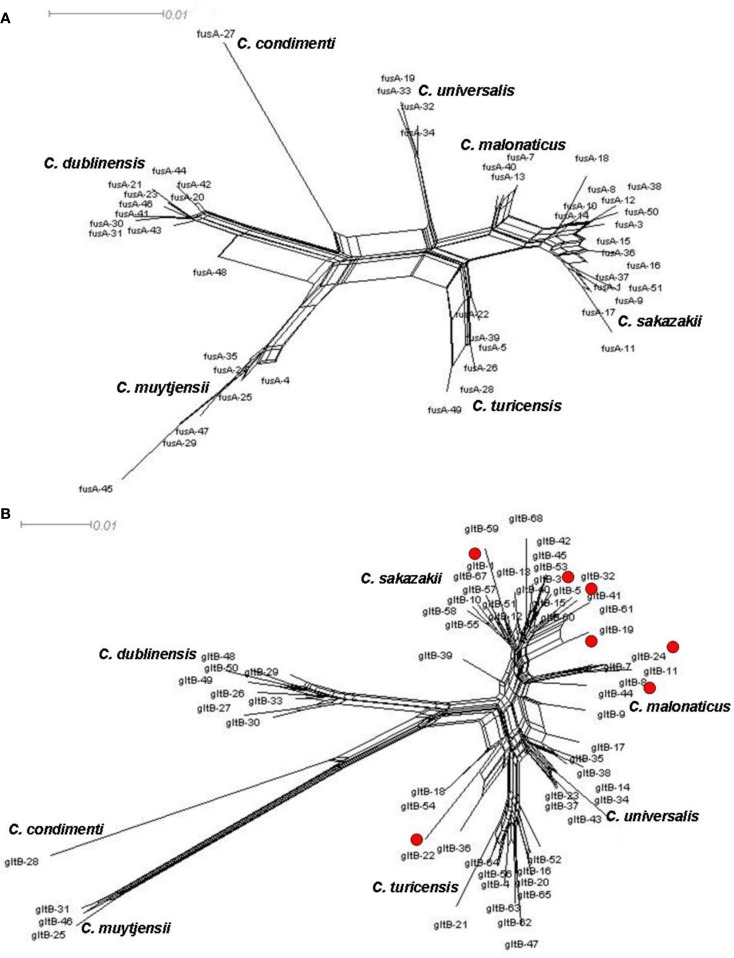
**Neighbor-net of allele sequence alignment for (A) *fusA* and (B) *gltB* generated for the *Cronobacter* MLST dataset indicating diversity and recombination events**. The figure has been drawn to scale using Splitstree4. The formation of parallelograms indicate possible recombination events.

There is evidence of homologous recombination (gene conversion) events having occurred in the evolution of the genus. This is covered in detail in Joseph et al. (2012b) and so only two loci, *fusA* and *gltB*, have been chosen to illustrate homologous recombination in *Cronobacter* spp. Figure 3 shows the neighbor-nets of these loci by Splitstree4 (Huson and Bryant, 2006) analysis to visualize recombination events and evolutionary relationships in the dataset. The formation of parallelograms in these figures denotes the possibility of recombination events. Among the seven loci, *fusA* is the most stable with the least number of shared alleles among species, and none of the profiles were shared between two or more species (Figure 3A). Thus *fusA* can be used for speciation of *Cronobacter* strains, and avoids the ambiguities of 16S rDNA sequence analysis. In contrast, there are some instances of allelic profiles being shared between two species. The *gltB* locus has the most (7) allelic profiles shared between species (Figure 3B). This sharing is mostly observed between *C. sakazakii* with *C. muytjensii* and *C. sakazakii* with *C. dublinensis* species. Despite these recombination events, the concatenation of the sequences of the seven loci for the MLSA helps to overcome any possible influence of this recombination and ensures a robust phylogeny for the genus.

Splitstree analysis also reveals a higher diversity in the *C. muytjensii* and *C. dublinensis* species than other *Cronobacter* species, with some individual branches being more genetically distant from the main cluster. This phenomenon has been described as “fuzzy” species in the MLST of *Neisseria* spp. (Hanage et al., 2005). Strain 1330 (ST 98) was one such candidate, seen as an lineage branching out from the *C. dublinensis* cluster which, following further phenotypic and DNA–DNA hybridization studies, was confirmed to be a previously unrecognized independent species, *C. condimenti* (Joseph et al., 2012a). Further recognition of new species in the *Cronobacter* genus is likely following MLST adoption, as the phylogenetic analysis is dynamic and not constrained to an analytical database.

Other divergent strains are evident in the *C. muytjensii* and *C. dublinensis* clusters and could indicate the presence of hitherto unrecognized *Cronobacter* species. The *C. muytjensii* species splits into two main clusters and a lone diverse branch (ST34). The *C. dublinensis* species shows greater diversity with two major clusters, and a few independent branches distant from each other. The diversity of these species has been investigated by Joseph et al. (2012b) using percent nucleotide divergence in the MLST sequence data, similar to the percent divergence used as cut-off thresholds for genus and species definitions using 16S rDNA analysis. Since the equivalent value for MLST has not been established, Joseph et al. (2012b) calculated the nucleotide divergence for the individual type strains of the *Cronobacter* species. The least divergence value was 2.8% (*n* = 3036 bp) which was between the type strains of *C. sakazakii* (NCTC 11467^T^; ST8) and *C. malonaticus* (CDC 1058-77^T^; ST7). This value was proposed as a minimum cut-off value for identifying potential candidates for new species. As a comparison these strains are only 0.3% different by full length 16S rDNA sequences (*n* = 1350 bp). The DNA divergence values between many strains in the *C. muytjensii* and *C. dublinensis* clusters were above this cut-off value. However, current taxonomic standards for recognizing new species by the *International Journal for Systematic and Evolutionary Microbiology* require a polyphasic analysis including DNA–DNA hybridization studies and phenotypic testing for biochemical traits. To date, our phenotypic profiling has not produced biochemical traits that could distinguish between the independent branches within *C. muytjensii* and *C. dublinensis* (Joseph, S., and Forsythe, S. J., unpublished data). The boundaries for defining a bacterial species have always been a topic of debate (Konstantinidis et al., 2006), and are ultimately a matter of taxonomic convenience. Some might argue about whether there is a real need for further delineation of already identified species. However, for an accurate understanding of an organism, it is very important to constantly evaluate the diversity of the organism at the genus and species level. From an evolutionary standpoint, this aids in an improved understanding of the relationships of the organisms with its possible closest ancestors. A whole genome level analysis should improve our understanding on this intra-species diversity, and certainly the need for subjective phenotypic tests to complement DNA sequence may mask further speciation.

## Clonality

*I*_A_ is a measure of the linkage of a population and when calculated using the MLST sequences the *I*_A_ values for the genus *Cronobacter* were found to be significantly greater than zero (Joseph et al., 2012b). This indicates the presence of linkage disequilibrium or clonality. This analysis has been highly significant for our understanding of both the diversity of the genus and the specificity of clinically relevant strains.

In this review article, the goeBURST algorithm in PHYLOViZ (Francisco et al., 2012) has been used to visualize the relatedness of *Cronobacter* STs. Primarily the diagrams show where STs differ in a one, two, or three of the seven loci, i.e., single locus variant (SLV), double locus variant (DLV), and triple locus variant (TLV) respectively. For example, ST4 and ST107 are SLV as they differ in the *fusA* allele; 5-1-3-3-5-5-4 and 5-50-3-3-5-5-4. It should be noted that this is not the same analysis of DNA sequence variation which has already been considered above under phylogeny and MLSA.

The goeBURST analysis of 115 identified STs for the *Cronobacter* genus at the time of analysis in the pubMLST database, shows there were 13 SLV clonal complexes (CC). Nine of these belong to *C. sakazakii*, two to *C. dublinensis* and one each to *C. turicensis* and *C. malonaticus*. *C. muytjensii*, *C. condimenti*, and *C. universalis* did not show the formation of any such CC. Apart from these SLVs, a number of DLVs were also observed and these are the STs which shared five out of the seven definitive allelic profiles. Some of these large CC are especially significant with respect to strain clustering according to their isolation sources. The reader should note that new STs are frequently added to the database and therefore such analysis should be revised in any presentations. For the purposes of this review only CC 1, 2, and 4 have been chosen for further discussion. The reader should refer to Joseph et al. (2012b) for a more detailed account.

Clonal complex 1 currently comprises *C. sakazakii* STs 1 and 14. ST1 is a dominant ST consisting of strains isolated from across the world over a period of more than 25 years. These have been mainly isolated from PIF and clinical cases, and also more recently from milk powder processing factories in Germany and Australia (Craven et al., 2010; Jacobs et al., 2011), apart from a few food isolates. ST14, which is a SLV (*ppsA* allele) of ST1 was isolated from infant formula (Caubilla-Barron et al., 2007). Also linked to this clonal complex is ST57, which is a DLV (alleles *atpD* and *glnS*) to ST1. This is the profile for a PIF isolate from Denmark in 1988, as reported by Muytjens et al. (1988) in their milk powder survey.

Clonal complex 2 comprises *C. malonaticus* STs 7, 84, and 89. ST7 is the predominant ST in this complex, and is a mixture of clinical and PIF isolates from over 30 years. STs 84 and 89 comprise clinical isolates from the Czech Republic. These clinical isolates were recovered mainly from fecal, sputum, and blood samples. Where information is available, these clinical strains were primarily from non-infants (i.e., >1 year in age), especially adults there are also food and weaning food isolates in this complex.

Clonal complex 4 comprises *C. sakazakii* STs 4, 15, 97, 107, and 108. This is a key complex with respect to *Cronobacter* spp. epidemiology. ST4 is the most dominant ST in this MLST study with 78 isolates, and also the most frequent clinical ST (Baldwin et al., 2009). However, the description of “clinical” when referring to the origin of strains is ambiguous. Such strains are not necessarily from the site of infection, i.e., conjunctivae swabs from meningitis cases, or even isolates from colonized asymptomatic individuals. Fortunately sufficient detailed information was available for these clinical *C. sakazakii* strains to reveal what is possibly the most significant finding generated by MLST analysis regarding the epidemiology and trophism of neonatal *Cronobacter* infections. Clonal complex 4 has been identified as a genetic signature for *C. sakazakii* neonatal meningitis, with the majority of the isolates being linked to meningitis cases over a period of 50 years from six different countries (Joseph and Forsythe, 2011; Hariri et al., 2012). Out of the 480 strains and 133 STs in the pubMLST database (data collected September 2012), 18 isolates are from the cerebral spinal fluid of meningitis cases. Of these, 16 are *C. sakazakii* ST4 and loci variants (STs 15, 97, 107, 108, and 110) belonging in the clonal complex 4. These include isolates from the 2011 highly publicized cases in the USA (Hariri et al., 2012). Why *C. sakazakii* clonal complex 4 predominates neonatal meningitis cases is presently unclear and could be due to environmental fitness factors as well as virulence traits. It is plausible that adult meningitis cases are unreported to date due to the maturity of the blood brain barrier.

Within the 15 2011 US strains, there were two ST4 SLVs. The CSF strain 1565 (ST107) differed from the ST4 profile in the *fusA* loci by 6/438 nt. Strain 1572 (ST108) isolated from an opened tin of PIF differed in the *fusA* loci by 5/438 nt. These two strains only differ from each other by 1 nt out of 3036 (concatenated length) in the *fusA* loci position 378 (A: T). This level of discrimination is not possible using PFGE. It should be noted that PFGE and MLST analyze the bacterial DNA content differently, and there are no *Xba*I sites (the endonuclease most commonly used with PFGE of Enterobacteriaceae) within the seven MLST loci.

Two strains which are not from the clonal complex 4 have also been associated with neonatal meningitis, both are ST1. The most well known being *C. sakazakii* BAA-894 from the publicized Tennessee outbreak (Himelright et al., 2002). This indicates that non-clonal complex 4 strains may on occasion also cause severe brain damage. The additional importance of strain BAA-894 is that it was the first *Cronobacter* to be fully sequenced (Kucerova et al., 2010, 2011). Also within the 2011 US isolates that have been profiled is a strain of *C. malonaticus* ST112. The strain had been isolated from the blood of a <1-month-old infant who died from a fatal case of meningitis. This isolate is highly significant as previously it had been observed that *C. malonaticus* predominates adult infections and no previous neonatal meningitis cases have been attributed to this species (Joseph and Forsythe, 2011).

In contrast to the correlation of clonal complex 4 with meningitis, no correlation of ST4 or other STs has been found with other clinical presentations such as NEC. *C. sakazakii* ST4 strains have been isolated from PIF collected in 12 countries. They have also been isolated from milk powder processing factories in Australia and Germany, including roller dryers, tanker bays, and spray dryers. Other non-clinical sources included isolates from weaning food, chocolate, and a washing brush (van Acker et al., 2001; Turcovský et al., 2011).

In addition to the three dominant CC of *C. sakazakii* and *C. malonaticus*, described above, some of the other SLVs and DLVs also showed clustering patterns depending on source such as clonal complex 8 (ST13 and ST86) from food and feed.

## Application of Multilocus Sequencing of *Cronobacter* spp. in Food Microbiology and Outbreak Investigations

A major impact of the multilocus sequencing of *Cronobacter* strains has been to support the recent definition of the *Cronobacter* genus. This has been essential for the evaluation of newly developed detection methods, and therefore of importance to the food industry and regulatory authorities for compliance. MLST has been used to profile *Cronobacter* strains from outbreaks in France, New Zealand, and the USA. This revealed the predominance of *C. sakazakii* ST4 in isolates from cerebral spinal fluid; Joseph and Forsythe (2011). More recently it was applied to *Cronobacter* strains submitted to CDC during 2011, and included the highly publicized cases last December. This confirmed the earlier study, by showing the CSF strains were *C. sakazakii* clonal complex 4 which includes ST4 (Hariri et al., 2012). The reason for this predominance is uncertain, but our studies of strains from the environmental sampling of milk powder processing factories, and also from PIF has also shown the predominance of the *C. sakazakii* species. Of particular interest is that *C. sakazakii* ST4 strains were isolated from powdered formulas as well as the roof, tanker bays, and roller driers of manufacturing plants. These dry environments may be selecting for desiccation resistant strains of *Cronobacter* spp. and in particular *C. sakazakii* ST4. In contrast, *Cronobacter* spp. isolates from a range of other foods, food ingredients, herbs, and spices are from a wide range of species and STs.

## goeBURST Analysis of *Cronobacter* spp. Distribution

**Figure 4:**
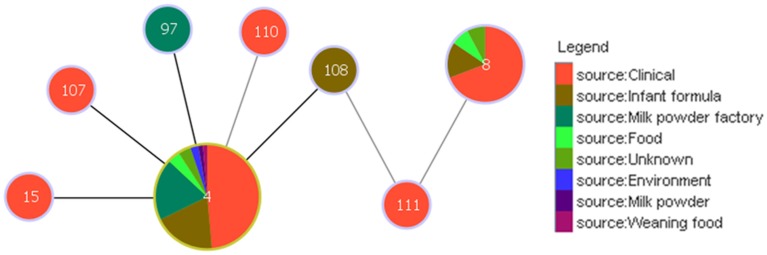
**Relationship between the clinically significant *C. sakazakii* ST4 clonal complex and ST8**. The threshold for the output was set to triple locus variation. The black lines denote the SLVs; while the gray lines indicate the TLVs. ST4 is the founder clone of the clonal complex.

Analysis using the goeBURST algorithm has revealed an interesting link between clonal complex 4 and ST8, via ST108 and ST111 with respect to the pathogenicity of *C. sakazakii*; Figure 4. ST8 comprises 13 strains, 9 of which are clinical isolates. Unlike ST4, none of these are associated with meningitis, but are associated with diarrhea. ST108 and ST111 were isolated from an opened tin of PIF and formula reconstitution water, respectively. Based on the goeBURST analysis, it is difficult to conclude whether ST8 could be a clonal descendant of ST4, since these are only indicative evolutionary relationships. However, the connection, albeit distant, does correlate with the epidemiology of a majority of the clinical strains of these STs.

**Figure 5:**
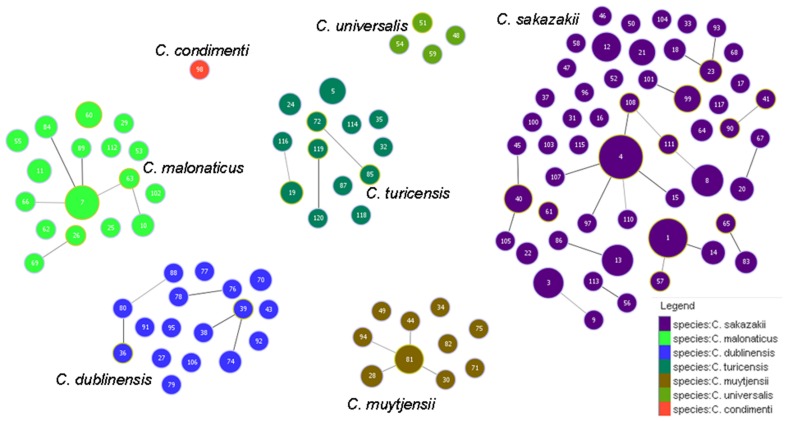
**Population snapshot of the *Cronobacter* MLST database generated using the goeBURST algorithm, indicating the clonal complexes and the breakdown of the species of the strains**. The threshold for the output was set to triple locus variation. The dominant STs are represented by the circles with larger diameters.

**Figure 6:**
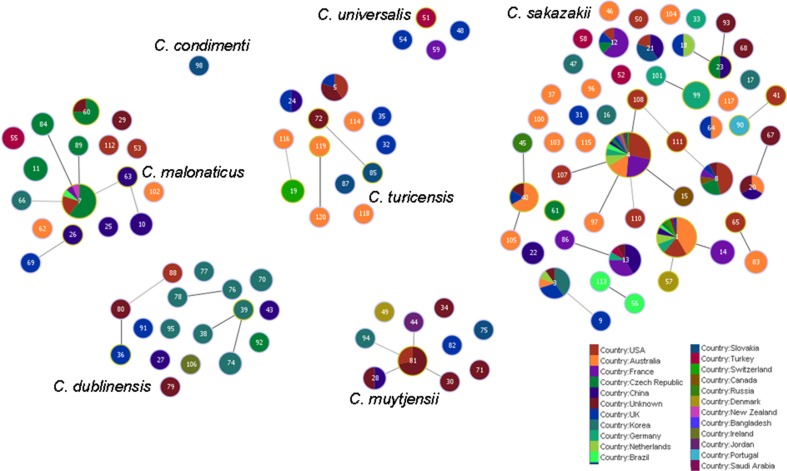
**Population snapshot of the *Cronobacter* MLST database generated using goeBURST, indicating the clonal complexes and the diversity of the strains based on country of isolation**. The threshold for the output was set to triple locus variation. The dominant STs are represented by the circles with larger diameters.

**Figure 7:**
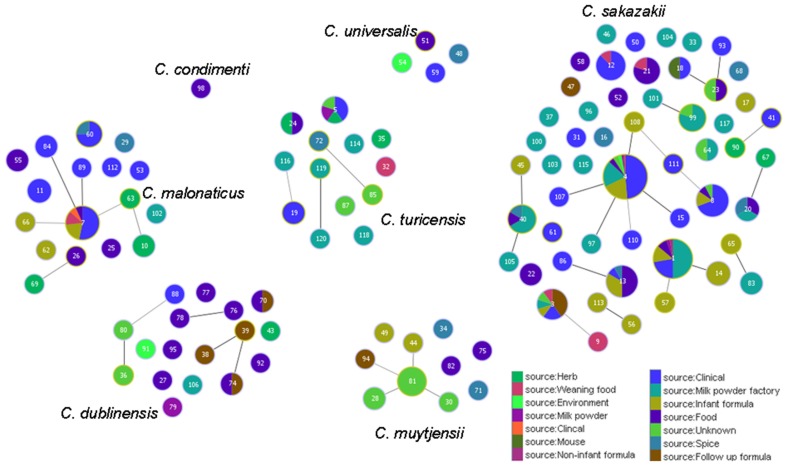
**Population snapshot of the *Cronobacter* MLST database generated using the goeBURST algorithm, indicating the clonal complexes and the diversity of the sources of the strains**. The threshold for the output was set to triple locus variation. The dominant STs are represented by the circles with larger diameters.

Figure 5 shows the goeBURST analysis of *Cronobacter* STs according to species. The formation of 13 SLV CC among 115 STs are revealed. The minimum requirement for the CC formation was an SLV linkage with the founder ST. Apart from these, a number of DLVs and TLVs were observed which, though not defined as CCs, have been indicated in the figures. Figure 6 shows the goeBURST analysis of *Cronobacter* STs according to the diversity of the countries of isolation. It illustrates the stability of certain STs such as ST1, ST4, and ST7 isolated from a range of countries over >50 years. Figure 7 is the goeBURST analysis of *Cronobacter* STs according to the diversity of their sources of isolation. This figure shows the majority of the clinical isolates to be concentrated in ST4, ST8, ST1, and their related STs, while ST1 seemed to also predominate with the milk powder factory and infant formula isolates. See also Table 1. Thus, the *Cronobacter* MLST scheme has been successful in the clustering of the clinically associated *Cronobacter* strains into specific STs/CC to form stable virulent lineages. A similar phenomenon has also been seen in the MLST of the meningococcus, *Neisseria meningitidis*, with the formation of hyper-invasive lineages (Caugant and Maiden, 2009).

**Table 1:** **Major clonal complexes in the *Cronobacter* MLST database**.

Clonal complex	Species	ST	No. of isolates	Isolation sources	Geographic distribution	Period of isolation
1	*C. sakazakii*	1	36	Clinical, PIF, formula, food, environment	UK, Australia, USA, Germany, China, Brazil, Czech Republic, Switzerland, Turkey, Russia, Netherlands	1979–2010
		14	3	PIF	France	1994
2	*C. malonaticus*	7	15	Food, clinical, PIF, weaning food	USA, New Zealand, France, Czech Republic, Brazil	1973–2007
		84	2	Clinical	Czech Republic	Unknown
		89	1	Clinical	Czech Republic	Unknown
4	*C. sakazakii*	4	78	Clinical, PIF, milk powder, weaning food, chocolate, washing brush, environment, prepared formula, foot wound	UK, USA, France, China, Canada, Netherlands, Germany, Russia, Czech Republic, Switzerland, Slovakia, New Zealand, Saudi Arabia, Bangladesh	1950–2011
		15	1	Clinical	Canada	2003
		97	1	Milk powder factory	Australia	2007
		107	1	Clinical	USA	2011
		108	1	PIF	USA	2011

*Adapted from Joseph et al. (2012b)*.

## Summary

The *Cronobacter* genus has come to the attention of the food industry, especially infant formula manufacturers, and regulators due to its association with life-threatening infections of neonates. Our current knowledge of the virulence and epidemiology of this organism is limited, and therefore an improved understanding of the diversity of the genus is warranted. Studying the organism through the application of MLST and MLSA has revealed a vast amount of information on the emergent bacterial pathogen, using just seven primer pairs with an costs equivalent to phenotyping and with all DNA sequence information being stored on an electronically portable open access database. This approach has established new *Cronobacter* species, the evolution of the genus and recognition of the clonal lineage attributed to the majority of neonatal meningitis cases. Supported by an open access curated database, the technique has become an established means of investigating an emergent bacterial pathogen which can cause a devastating infection in newborn babies. As an example of the benefits of rapid DNA sequencing, even without considering Next Generation Sequencing of genomes, the study of just seven loci is a model for modern microbial epidemiology and food microbiology.

## Conflict of Interest Statement

The authors declare that the research was conducted in the absence of any commercial or financial relationships that could be construed as a potential conflict of interest.
